# Review of the Upright Balance Assessment Based on the Force Plate

**DOI:** 10.3390/ijerph18052696

**Published:** 2021-03-08

**Authors:** Baoliang Chen, Peng Liu, Feiyun Xiao, Zhengshi Liu, Yong Wang

**Affiliations:** School of Mechanical Engineering, Hefei University of Technology, Hefei 230009, China; baoliang2017@mail.hfut.edu.cn (B.C.); pengl0201@163.com (P.L.); xfymusic@163.com (F.X.); zslhfut@hfut.edu.cn (Z.L.)

**Keywords:** posture balance, balance assessment, force plate, COP

## Abstract

Quantitative assessment is crucial for the evaluation of human postural balance. The force plate system is the key quantitative balance assessment method. The purpose of this study is to review the important concepts in balance assessment and analyze the experimental conditions, parameter variables, and application scope based on force plate technology. As there is a wide range of balance assessment tests and a variety of commercial force plate systems to choose from, there is room for further improvement of the test details and evaluation variables of the balance assessment. The recommendations presented in this article are the foundation and key part of the postural balance assessment; these recommendations focus on the type of force plate, the subject’s foot posture, and the choice of assessment variables, which further enriches the content of posturography. In order to promote a more reasonable balance assessment method based on force plates, further methodological research and a stronger consensus are still needed.

## 1. Introduction

Injuries due to falls represent a major worldwide public health problem [1,2,3,4]. As per the data in the World Health Organization (2008) report, approximately 28–35% of people aged ≥64 years experience falls every year [5]. The most serious injuries caused by falls include brain trauma and hip fractures, the former accounting for 46% of fatal deaths among the elderly [6]. Extensive and in-depth research on the mechanisms and risk factors of falls has revealed that they are strongly associated with balance disorders and abnormal gait [7,8].

Balance ability is a key skill for avoiding falls [9,10]. Being able to maintain an upright balance without falling is the primary condition for independent activities of daily living [11,12,13,14]. Reviewing the literature, we can see that the summary and reflection on the evaluation methods of postural balance are in progress. For example, reference [15] reviews the functional and physiological evaluation of balance, and concludes that the functional evaluation method can be used as a screening tool to identify subjects who need further comprehensive balance information acquisition, and further indicates that the dynamic force platform can provide information about multiple components of the posture control system under dynamic conditions, which can quantify subtle changes in balance ability. Reference [16] discusses the variables in the balance assessment methods of static and dynamic force plates that can predict falls in the elderly. Reference [17] reviews the application progress and usage methods of accelerometers in children’s postural balance assessment.

For evaluating balance function, there are often certain differences in evaluation results among studies. On the one hand, these differences are caused by various measurement and evaluation techniques used in different professional fields. On the other hand, these differences are related to the inconsistent protocols (i.e., the selection of test equipment, the number of trials and the length of time, the posture of the feet, and the selection of assessment parameters) of postural balance assessment [18,19]. The purpose of this article is to review the main concepts and methods used in postural balance assessment, summarize the key items in the assessment test that will affect the assessment results, analyze the variables in the force plate balance assessment method, and provide valuable reference suggestions for the research in this field.

## 2. Balance Control

A seemingly simple and common body movement involves a complex balance mechanism. Balance control requires the coordination of different subsystems [20,21]. These subsystems include “anticipatory posture adjustment” (APA), “reactive posture adjustment” (RPC), “dynamic gait” (DG), “sensory orientation” (SO), biomechanical constraints, information processing, etc. These subsystems mainly come from three categories: biomechanics, motor coordination, and sensory organization; each subsystem comprises specific neurophysiological mechanisms [11], but each task may involve multiple subsystems, and there is an interaction [22]. APA represents the ability to make active posture adjustment before voluntary movement and to transfer the center of mass; RPC represents the ability of the body to react passively to the posture after an external perturbation and to recover the center of mass to a stable range; SO indicates the ability to orient various segments of the body based on the various conditions of gravity, the support surface, visual surround, and internal references [20]; and DG refers to the ability to continuously shift the center of mass and control postural stability when the base of plantar support is changing [22].

The biomechanical constraints system is the basis of the clinical assessment of balance, including not only joint and muscle function, but also strength, joint range of motion, flexibility, geometric posture alignment of multiple body segments, stability limits, etc. The stability limit refers to the maximum range that the body’s center of gravity can be moved in different directions [9]. The information processing system of balance control is the sorting, processing, weighting balance sensory information, and selecting the appropriate motion response [9,20]. In the case of complex environments and multiple tasks, the amount of information will increase. Information exceeding a certain limit may conflict with the limited central processing capacity, which will affect balance control. For example, frail elderly people or patients communicating with others while walking will impair balance control.

## 3. Center of Pressure (COP) and Center of Gravity (COG)

The center of mass (COM) is an imaginary point, which is a concentrated expression of the overall weight, which can be calculated as the weighted average position of each segment of the body in the three-dimensional space [23,24]. The vertical projection of COM on the ground is usually called COG (center of gravity) [23]. The center of pressure (COP) is defined as the point where the plantar ground reaction force is applied, and it represents the center point of the entire pressure in the foot–ground contact surface [25,26,27]. The ground reaction force is generally measured by a force measurement platform. COM is a passive variable controlled by the posture control system, while COP is a comprehensive control variable, which is the result of the inertia force of the sway body and restoring equilibrium forces of the posture control system [23,24]. The concepts expressed by COP and COG are different; only in the static upright posture, they may present similar variations. The difference between COG and COP that characterizes posture is related to body acceleration; greater body sway frequencies result in an increase in the difference between these two variables [28].

**Figure 1:**
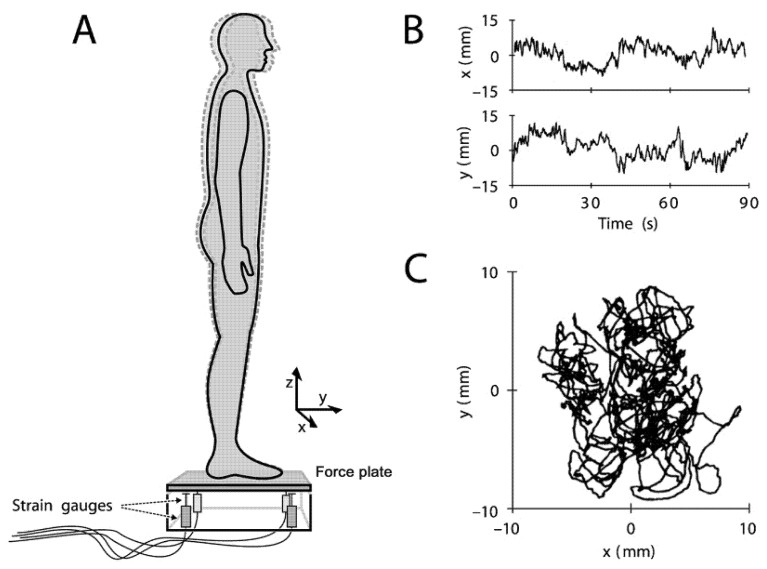
Change of the center of pressure (COP) calculated through the force plate. (**A**) Stand statically on the force plate for the postural sway test. The dotted line of the humanoid outline indicates spontaneous sway. The force plate is usually arranged with strain gauges at the four corners below the supporting plate, which can calculate the ground reaction force and COP trajectory. (**B**): COP time series in ML(x) and AP(y) directions. (**C**): The trajectory of COP [31].

Parameters derived from the COP trajectories obtained by a high-precision force plate are considered to be the gold standard of balance performance [29,30]. There are two types of commonly used force plates: (1) uniaxial force plates equipped with a single-axis load cell that measure only the vertical component (F_Z_) of the ground reaction force and (2) force plates equipped with load cells (strain gauges or piezoelectric sensors usually arranged on the four corners of the plate), which can obtain the three components, F_X_, F_Y_, and F_Z,_ of the ground reaction force and the moment of force acting on the plate. Moments M_X_, M_Y_, and M_Z_ are called multi-axis force plates. Both uniaxial and multiaxial plates can be used to calculate the medial–lateral (ML) and the anterior–posterior (AP) time series of COP over time during a task of postural test (Figure 1).

**Figure 2:**
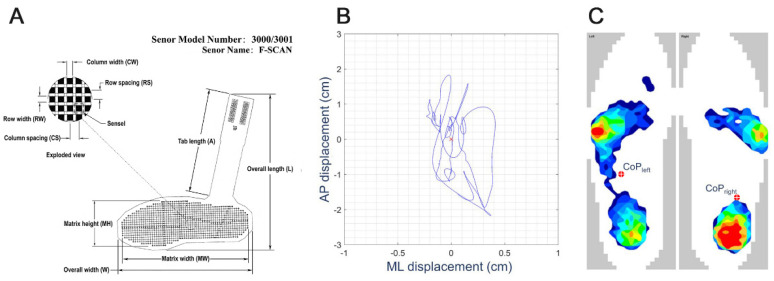
(**A**) Smart insole introduced by Tekscan, Inc. It is an insole with a series of pressure sensors; the resistance of the sensor will change with applied pressure [33]. (**B**) COP displacement traced using the smart insole system [14]. (**C**) Representative bi-plantar pressure map during upright standing. The red circle represents the left and right plantar COP calculated from the pressure matrix [14].

Pressure measurement platforms or smart insoles with a large number of built-in pressure sensors (Figure 2A) can also be used to obtain the plantar COP displacement (Figure 2B) [29]. The embedded in-shoe system can measure the pressure between the foot and the shoe [32]. Through the voltage value change matrix obtained by a large number of built-in pressure sensors, the computer expresses different pressure values via different colors (Figure 2C), which can more intuitively display the pressure distribution information of the sole [33]. The main limitation of the in-shoe system is that the sensor performance will be reduced with repeated experiments. Additionally, the sensor may slip during use. Furthermore, heat and sweat may also affect the results [32].

The measurement and acquisition of COG is particularly meaningful, especially in understanding the relationship between COG and COP [34]. The first method of calculation utilizes kinematics equipment. The three-dimensional (3D) motion capture system can provide accuracy and reliability by recording subtle motions [35]. The most commonly used method is to use high-resolution and high-speed cameras to capture the reflection markers placed on specific anatomical locations, and to combine with software to identify these positions. When estimating the specific position of COG, an accurate human body model is required; the instantaneous position of each segment of the body is combined with inertial parameters to determine the COG of the body [36]. This type of method requires a high-precision 3D motion capture system and a marker to be attached to the subject’s body joints, and thus presents high requirements for space, time, cost, and personnel [35,37]. In addition, the inertial parameters of various parts of the body have considerable errors [18]. In a second method, the horizontal component of the COG can be estimated by a double integration of the horizontal force divided by the mass. How to find the initial position and speed of the body is the primary difficulty of this method [18]. The third method evaluates the COG horizontal position using the inverted pendulum model and the filtering method based on the relationship between the COG and COP in the frequency domain from the COP displacement measured from a single-axis or multi-axis force plate [34]. Among the three methods, only kinematic analysis can calculate COG motion in three spatial directions.

## 4. Standardization of Experimental Conditions for Balance Assessment

The assessment of human standing balance is usually achieved via spontaneous sway and induced sway of posture [38]. The force plate can perform static upright posture and dynamic balance assessment and can also combine disturbance devices and technology to evaluate reactive balance. Static balance assessment generally requires the subject to stand on a stable support surface and keep the COP within this support surface; actions or conditions that increase the difficulty of maintaining balance can be added during the assessment, such as standing on one foot, standing with eyes closed, or standing on an elastic cushion. In the autonomous posture conversion balance assessment, the subjects perform body movements or actions while keeping the support surface stable; these movements commonly include sitting-to-stand transfer [39], squatting [40], and switching between standing on two feet and standing on one foot [41,42]. A reactive balance assessment is usually an induced sway balance test using a mobile platform [43]. A comparison of the literature shows that there are many differences in experimental conditions when using a force plate for balance assessment, and some of these differences will have a great impact on the results. Therefore, it is necessary to review the experimental conditions of the balance assessment. Standardized experimental conditions can reduce the influence of confounding variables on the balance assessment, which is beneficial to the clinical management of the patient’s balance assessment.

First, conditions in the assessment environment. It is necessary to ensure the consistency of environmental conditions such as good lighting, silence and no noise, and comfortable temperature (except for these variables as research purposes), The special environment will affect the subject’s posture control subsystems, such as the SO and information processing systems mentioned above. In the specific balance evaluation research, we try to eliminate confounding factors that may affect the evaluation structure [20,34,44].

Second, because COP is the result of the muscle action of the two feet, it is necessary to use two independent force plates placed under the left and right foot to measure the ground reaction force and COP under each foot, which can be more objective than using a single force plate to analyze the balance mechanism [23,45]. This is especially important for people with asymmetric weight distribution, such as those with hemiplegia and amputation patients [46].

Third, the literature reports different test durations for evaluating posture control as well as a varying number of trials [34]. Generally, the test duration under static posture task is longer than that under dynamic posture task [34]. Under static conditions, since the steady process of posture control (the stationarity of the posture signal) requires a few seconds of adjustment time [47], the International Posture and Gait Research Society recommends that the parameters are stable and reliable within the recording time of 25–40 s [48]. It is important to ensure that the duration of the test does not cause fatigue, especially in subjects suffering from pathology [34]. It is worth mentioning that in the documents [49,50,51], using a 30-min long-term posture task, the subject can choose the posture of the foot and adjust the posture without restriction (except the requirement not to step off the force plate); researchers believe that the balance test in the natural state can obtain valuable information. Under dynamic conditions, the duration of each trial should be between 15 and 25 s for healthy subjects, and for subjects with a weaker physical condition, a duration of 10–20 s is more appropriate [34]. The reactivity balance assessment requires little time; it only needs to analyze the data for a few seconds before and after the disturbance to assess the postural control of the subjects [38,52,53]. It is important to remind that insufficient test duration may lose data information, and too long test duration will increase fatigue factors, thereby affecting the accuracy of certain evaluation variables. Therefore, it is necessary to standardize the test duration range for different subjects and task types. Regarding the number of trials, it should be noted that repeating the same posture task continuously may lead to learning effects and a gradual reduction in postural sway. Conversely, performing the same task multiple times may cause fatigue and increase the postural sway. It is recommended that if there are multiple test tasks in the assessment, the order of the tests should be arranged randomly to avoid the learning effect and fatigue caused by multiple identical tests [52].

Fourth, foot posture directly affects the area of the support foundation; thus, it is critical to ensure the standardization of the foot posture among different subjects and multiple trials of the same subject for the postural balance assessment. Several studies suggest that the opening angle of the inner side of the left and right foot and the inner distance of the heel are defined to form a standardization related to the posture of the foot. However, this uniformly prescribed foot posture does not consider the specific characteristics of each subject and may cause some subjects to complete the test in an unnatural posture state, leading to deviations in the evaluation results [54,55,56]. It may be a better option for subjects to use their most natural foot posture for balance assessments.

Fifth, conducting the postural balance assessment with disturbances may cause the subjects to lose their balance. Safety during the assessment is very important [18], and safety systems are implemented to avoid falls caused by loss of balance [57,58,59]. It is worth noting that the tactile cues imparted by the seat belt may affect the assessment of postural balance [60]. Previous research reports claim that when the subject’s body is briefly and lightly touched with objects outside the body, the postural sway can be reduced [61,62]. The safety system produces a light touch effect when it comes into contact with the body, which may add somatosensory information and spatial reference for the posture control system, resulting in a reduction in postural sway. Therefore, when using a safety system, it is necessary to set the upper limit of the contact force value to avoid providing excessive mechanical support.

Sixth, subjects are usually asked to stare at a target in space during the postural balance assessment. The distance between the eyes and the target will affect the stability of the posture. For example, compared to a distance of 3 m, subjects have reduced postural sway when the target is positioned 40 cm from the subject’s eyes [63]. Usually, the target is placed at a height equivalent to the subject’s line of sight and at a distance between 1–3 m.

## 5. Common Parameters in Balance Measurement Based on the Force Plate

The force plates are widely used in posture balance assessment; ground reaction force and COP are the main output parameters of the plates. The original COP signal can be expressed in two ways, namely, the statokinesigram and the stabilogram. The state motion diagram illustrates the real-time presentation of COP displacement on the horizontal plane, and the stability diagram represents the amplitude changes of COP in the AP and ML directions, respectively. Other COP variables can be calculated based on the raw COP data to evaluate the posture function [34]. However, there is no consensus on which variables of COP should be extracted for the postural balance assessment [64,65].

COP-based variable analysis can be divided into two categories: global variables and structural variables. Global variables are the size of COP parameters in the time and frequency domain. It is generally believed that the large related COP parameters in global variables result in poor posture stability. Structural variables are the decomposition of COP data into sub-units and with the motion control process; thus, a more detailed analysis of COP time evolution could be performed [18,34,66]. Global variables lack the expression of changing structure, and structural variables may provide important information on the process of posture control [18,34]. Because the variables of COP can be derived from many kinds of COP variables, it is necessary to combine the characteristics of specific postural tasks to select more suitable variables for balance assessment.

The following introduces some common variables in COP signal analysis. It is worth noting that the assessment results of each patient should be interpreted in conjunction with other clinical assessments (for example, motor recovery, sensation, and cognition) [37].

First is the COP ellipse area, wherein the ellipse set of the data is used to quantify 90% or 95% of the total area formed by the COP trajectory covered by the AP and ML directions. It is regarded as an indicator of the overall posture performance, and it is generally believed that the smaller the area, the better the postural balance. However, the small area caused by too-narrow COP displacement may indicate that the posture control is too rigid (called “freezing”), which may reduce the ability to adapt to external challenges [14]. The elliptical area of COP represents quantified time information of posture stability and is considered an important part of posturography research [57,67].

Secondly, the COP path length is the total distance traveled by COP during the test time [68]. The smaller the path length, the better is the postural stability. It is an effective evaluation index in numerous populations and balance conditions.

The third variable discussed is the COP amplitude, which is the distance between the maximum and minimum COP peak points in the AP and ML directions. Large amplitude value is due to poor postural stability. COP amplitude is a commonly used and reliable parameter that has been widely used in balance assessment. In the ML direction particularly, it has good reliability and validity in the evaluation of patients with neuromotor disorders [34,69]. The prerequisite for selecting COP amplitude as the evaluation variable is to ensure the accurate positioning of the subject’s foot posture in the AP and ML directions on the force plate during the evaluation. As mentioned above, the uniformly prescribed foot posture for all subjects may lead to evaluation results in abnormal postures, which will lead to conflicts in the choice of foot posture. Our suggestion is that the balance assessment under self-selection/natural posture is a better choice. Therefore, it is necessary to make a complete definition of the subject’s biped posture after choosing, and use the parameters defined by the foot posture to construct a coordinate system for calculating COP, instead of using the coordinate system of the force plate to locate the subject’s foot posture. The COP track obtained in this way contains the position information of the foot posture, and it is more accurate to calculate the COP amplitude in different directions. This evaluation method requires the force plate system to be able to cooperate with the subject to adjust the foot posture flexibly. No relevant literature reports have been found so far.

Fourth, the average COP speed is calculated by dividing the COP path length by testing time. In addition to the total COP average velocity, the average COP velocity components in the AP and ML directions can also be calculated. COP speed is an indicator of the efficiency of the postural control system. The average COP speed value is small, which is considered a good performance of posture control. Among the COP parameters of patients with different age groups and different neurological diseases, COP speed is the most sensitive parameter [70].

Fifth, the standard deviation and root mean square (RMS) are variability indexes of COP motion track that show good discrimination ability in different age groups and pathology subjects. Due to the inherent relationship between COP and COG, a high COP RMS indicates a poor posture control [37]. A study reports that an increased RMS in the ML COP is related to an increased chance of falling in patients who suffered a stroke [71].

Sixth, the Romberg quotient is the ratio of the eyes-closed to the corresponding eyes-open measure [68]. In Romberg test, the subjects were asked to stand with eyes closed and keep balance while eliminating the information from the visual system. Romberg quotient can be used to evaluate whether there is impairment of the proprioceptive and vestibular system [28]. Normal value of Romberg quotient is >1, but values that far exceed 1 indicate that the patient is dependent on visual information to provide sensory information for control balance. The research report stated that the upper limits of AP and ML of the 95% confidence interval of the elderly Romberg quotient are 1.3 and 1.1, respectively, which are within the normal range, and there is no increase in the risk of falling. If it is higher than this value, it may indicate that the balance function is impaired [37].

Seventh, the symmetry index is a parameter that measures the contribution of each limb to the AP balance control and is expressed by dividing the RMS of AP COP value on the advantageous side by the sum of the RMS of AP COP value on both sides [72]. A symmetry index equal to 0.5 means that the contribution of the two limbs are equal; >0.5 indicates that the advantageous limb has a large contribution to balance control, and <0.5 indicates that the disadvantageous limb has a large contribution to balance control. Symmetry index can predict the risk of falls in daily life of patients who suffered a stroke [37].

Eighth, weight-bearing asymmetry is a measure of the weight distribution between the lower limbs. It is expressed as the percentage of the average vertical force under the limbs with greater force to the total weight. A stroke patient may stand asymmetrically due to impaired sensorimotor on the affected side or an eccentric frame of references [20,37]. The height asymmetry of weight-bearing when standing quietly is related to the decrease in walking speed and the greater asymmetry in walking time and space characteristics of stroke individuals [37].

Ninth, sample entropy (time series complexity measure, which can quantify the regularity or predictability of time series, proposed by Richman and Moorman in 2000) [66,73], approximate entropy (a measure of unpredictability), and Lyapunov exponent (a measure of difference) are parameters used to analyze the COP structure. Patients with neurological diseases usually show lower values of SampEN, ApEn, and LyE, indicating that the efficiency of posture control is lower, and the adaptability and responsiveness of anti-interference are reduced [74,75].

The above COP variables are mainly suitable for static balance and dynamic balance assessment. In a reactive balance assessment, a unified standard is difficult due to the diversity of the dynamic interference technologies. The dynamic balance disturbance test is presented in reference [38], because the automatic response part of the posture control system is the focus of research, and the amplitude of the first peak in the COP trajectory and the time required to reach the first peak are selected. In reference [76], the author selected three variables (COG displacement, COP displacement, and COG–COP distance) with the balance board on the surface of the force plate to investigate the posture control adaptation of the balance board. In reference [77], the study assessed the effect of lateral perturbations on postural re-stabilization regarding lower limb preference, The COP peak displacement, the displacement from the first peak to the second peak, the time from the start of perturbations to the first peak, the time from the first peak to the second peak, and the time of re-stabilization were selected as the evaluation variables. Reference [53] used the computerized dynamic posturography and the virtual reality system to evaluate the postural control ability across the aging process. In the continuous time section of slip forward, pitch down, pitch up, and re-stabilized, the COP displacement in the AP and ML directions and the WB ratio are selected as the evaluation variables. The WB ratio is determined by the GRF of the left limb divided by the GRF of the right limb. Reference [78] used COP_D_ and COP_V_ as the variables of postural sway in the unilateral posture balance task, where COP_D_ is the difference between the COP peak position and the COP position at the beginning of the perturbations, and COP_V_ is COP_D_/t (t is from the beginning of the disturbance to time interval between peak time points). Reference [79] studies the automatic posture response in the three stages of fatigue (before fatigue, after fatigue, and after recovery), and selects peak COP displacement and peak COP speed as part of the evaluation variables.

## 6. Discussion

Balance assessment methods can range from clinical balance assessments based on observational and scale methods to laboratory measurements based on kinematics and kinetics [80]. Observation methods include those such as the Romberg experiment. The Romberg experiment compares the subjects’ balance when standing with eyes open and closed. It can screen and diagnose proprioceptive dysfunction [81,82]. The scale method is a combination of a series of posture tasks closely related to daily life to score and count the performance of each subject, and compare the score interval criteria to perform a balance assessment. Commonly used scales include Berg Balance Scale [83], The Tinetti Performance Oriented Mobility Assessment (POMA) [84], dynamic gait index (DGI) [85], etc. Different scales generally target different potential patients with balance disorders. BBS may be the most commonly used balance indicator [86] in stroke rehabilitation. Tinetti POMA aims to measure the balance and gait function of the elderly [84]. The DGI was developed by ShumwayCook and Woollacott to assess the functional stability and fall risk of the elderly [85]. In addition to the elderly, DGI has also been used to evaluate balance disorders in patients with Parkinson’s disease [87], multiple sclerosis [88], vestibular dysfunction [89], and other diseases. Non-instrumental tests such as observation method and scale method have upper limit effect, low sensitivity, and subjective evaluation [80,90,91].

Non-instrument tests in balance assessment cannot detect subtle changes in posture control and only provide a gross indicator. Such methods have a limited ability to provide potential mechanisms for balance control and biomechanical defects related to balance damage [37,57,90,92]. Therefore, quantitative assessment of balance is essential, especially for technical methods that are convenient for clinical use. Objective and quantitative balance assessment is regarded as the key to disease tracking and therapeutic intervention. COG and COP are crucial parameters for balance assessment, which can be reflected and analyzed to assess body sway and postural sway, respectively. This paper analyzes the relevant details that affect the evaluation results in the balance assessment based on the force plate. This focus includes the choice of the type and number of force plates, the subject’s foot posture, and the choice of assessment variables.

**Figure 3:**
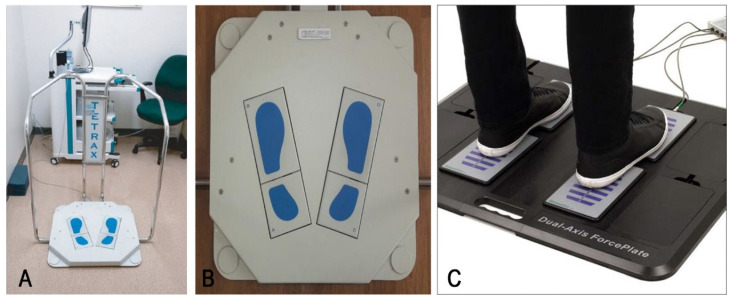
(**A**) Tetrax ^®^ balance test system (sunlight, Israel), (**B**) Tetrax ^®^ balance test system bottom force plate structure, (**C**) Biometrics’ ForcePlates balance system (Biometrics, UK).

The COP trajectory obtained by a single force plate is the sum of the spatiotemporal effects caused by the postural changes of AP and ML [45]. It is affected by the simultaneous control of the hip and ankle joints, but it is often understood as the result of the control of the ankle muscles. The use of two force plates placed under the left and right feet for an upright balance test can better distinguish the hip joint mechanism from the ankle mechanism and is more conducive to the assessment of the asymmetry of the left and right sides [40,93,94]. Due to the existence of human foot arch and mechanical structure characteristics [95], the main stress parts of the foot are the sole and the heel [96]. Some force plate systems are equipped with independent force plates on the heel and sole of a single foot. More ground reaction force distribution information on the foot, including the force data from the left and right heels and soles, can be used to evaluate the interaction and changes between sides to obtain more detailed human posture parameters. Such commercial force plate systems include the Tetrax balance test system (sunlight, Israel) and the ForcePlates balance system (Biometrics, UK) (Figure 3). In addition, this two-zone method can conveniently place the longitudinal arch on the gap between the front and rear force plates so that the positioning of the foot is standardized and the analysis variability is reduced.

In study [97], among 181 elderly and 81 young people, the average angle of the optional foot position during the bipedal position was 15.1° abduction (range 13° to 52°). Such results highlight lack of standardization of the universal balance assessment and are of particular concern because the posture of the feet affects the balance of the bipedal standing state. Due to the connection between single-leg and double-leg balance performance, standardized foot posture is also critical for single-leg posture tasks [13]. Therefore, in the study of bipedal or single-footed standing balance assessment, foot alignment settings are required [25]. However, it is worth noting that the newly set foot posture may change the subject’s habitual posture, leading to results obtained only under certain conditions and cannot objectively represent the subject’s true balance ability [98]. It may be a better choice to let subjects adopt their natural posture. At the same time, postural stability will also have an important correlation with height and foot size (mainly foot length and foot width), and assessment variables can be normalized with appropriate anthropometric characteristics [18,99]. For example, in the literature [53], COP variables are standardized to the length and width of the foot to minimize the influence of anthropometrics.

In addition to the commonly used evaluation parameters summarized in the article, Blaszczyk [64,65,100] proposed three new evaluation parameters: sway direction index, sway ratio (SR) and sway vector (SV). The author believes that they are independent of COP sampling frequency and the length of a trial and suggested that SV may be a useful standard in static balance assessments. Blaszczyk stated [101] that an increased SR may be the effective predictor of postural deficit and falling risk in Parkinsonians.

## 7. Conclusions

The force plate is currently a widely-used method and technology for quantitative postural balance assessment in laboratories and clinics [102]. This article attempts to explain the importance of the standardization of experimental conditions based on dynamometer, the analysis method of posture balance, and the selection of variables in balance evaluation. Consistency of environmental conditions, the use of dual force plates, natural foot posture to prevent fatigue caused by long time or high difficulty, and the postural tasks of subjects with balance disorders need to transition from simple to complex. These are suggestions that can reduce the impact of confounding variables. The selection of assessment variables should be determined in conjunction with the specific assessment tasks. The choice of statically balanced COP variables is consistent and generally includes COP area, COP average speed, COP RMS, and COP amplitude. Variable selection in the self-dynamic balance assessment focuses more on the symmetry and variability of the two sides. Finally, there is greater flexibility in the variable selection for the reactive balance assessment, and variables that focus on the time of the automatic posture reaction stage and the peak response are desirable.

The posture control theory included in the balance assessment is very extensive. The suggestions proposed in this article are the foundation and key part of postural balance assessment. In order to promote a more reasonable balance assessment method based on force plates, further methodological research and a stronger consensus is required.
